# Observing Changes in Ocean Carbonate Chemistry: Our Autonomous Future

**DOI:** 10.1007/s40641-019-00129-8

**Published:** 2019-05-07

**Authors:** Seth M. Bushinsky, Yuichiro Takeshita, Nancy L. Williams

**Affiliations:** 1 Program in Atmospheric and Oceanic Sciences, Princeton University, 300 Forrestal Road, Sayre Hall, Princeton, NJ 08544 USA; 2 Monterey Bay Aquarium Research Institute, 7700 Sandholdt Road, Moss Landing, CA USA; 3 Pacific Marine Environmental Laboratory, National Oceanic and Atmospheric Administration, 7600 Sand Point Way, NE, Seattle, WA USA

**Keywords:** Autonomous platforms, Carbonate observations, Ocean acidification, Ocean biogeochemical sensors

## Abstract

**Purpose of Review:**

We summarize recent progress on autonomous observations of ocean carbonate chemistry and the development of a network of sensors capable of observing carbonate processes at multiple temporal and spatial scales.

**Recent Findings:**

The development of versatile pH sensors suitable for both deployment on autonomous vehicles and in compact, fixed ecosystem observatories has been a major development in the field. The initial large-scale deployment of profiling floats equipped with these new pH sensors in the Southern Ocean has demonstrated the feasibility of a global autonomous open-ocean carbonate observing system.

**Summary:**

Our developing network of autonomous carbonate observations is currently targeted at surface ocean CO_2_ fluxes and compact ecosystem observatories. New integration of developed sensors on gliders and surface vehicles will increase our coastal and regional observational capability. Most autonomous platforms observe a single carbonate parameter, which leaves us reliant on the use of empirical relationships to constrain the rest of the carbonate system. Sensors now in development promise the ability to observe multiple carbonate system parameters from a range of vehicles in the near future.

## Introduction

The oceanic carbonate system is going through unprecedented change. Each year, the ocean absorbs approximately 25% of anthropogenic emissions of carbon dioxide (CO_2_) to the atmosphere [1] and has absorbed at least 25% of all anthropogenic CO_2_ since the industrial revolution [2, 3]. While this reduces atmospheric CO_2_ concentrations, it comes at a cost. The dissolving of CO_2_ acidifies the seawater (lowers pH) and shifts the equilibrium of carbonate species, decreasing carbonate ion and increasing bicarbonate concentration [4–6]. On average, open ocean pH has decreased by approximately 0.0018 year^−1^ over the past 15–30 years [7]. This process, known as ocean acidification, is happening more rapidly than at any other time in Earth’s history [8].

Ocean acidification is thought to have widespread detrimental impacts on marine organisms and ecosystems including those that support valuable fisheries [9–11]. For example, pteropods, a pelagic sea snail that is an important prey species for fish such as salmon, cod, and mackerel, have been demonstrated to be especially vulnerable to elevated CO_2_ conditions [12]. Mass mortality events in shellfish hatcheries have also been linked to ocean acidification [13]. Coral reefs, which provide trillions of dollars in societal services worldwide, are projected to experience decreased net calcification, a key process in maintaining ecosystem function [14]. The impact of acidification is being felt globally, but with significant heterogeneity in the temporal and spatial patterns of response due to regional differences in chemistry, circulation, and biology. For example, modeling results predict that some ocean regions will acidify significantly faster than the open ocean, such as upwelling regions like the California Current System [15], the Arctic Ocean [16], and the Southern Ocean [17], making them potentially more vulnerable to ocean acidification. To observe predicted changes in the ocean carbonate system, we must have an instrumentation network that can capture acidification and its effects at multiple temporal and spatial scales.

There are four “master” variables for the marine carbonate system that we can measure: partial pressure (or fugacity) of CO_2_ (*p*CO_2_), pH, dissolved inorganic carbon (DIC), and total alkalinity (TA). The carbonate system can be described by a system of equations such that it can be fully constrained by measuring any two of the four parameters [4, 5]. Different combinations of parameters must be measured or calculated depending on the biogeochemical processes of interest. For example, *p*CO_2_ is required to study the air-sea CO_2_ flux, as the difference between air and seawater *p*CO_2_ describes the thermodynamic potential for CO_2_ to go in or out of the water [18]. DIC is particularly useful for studying production and respiration dynamics in the open ocean [19, 20]. TA can be used to study calcification and dissolution processes. Anthropogenic carbon inventory calculations often use both DIC and TA [21].

Observing changes in ocean conditions on the spatiotemporal scales necessary to constrain carbon uptake, storage rates, and subsequent ecosystem impacts remains a significant challenge. Prior to the development of autonomous systems to measure ocean carbonate chemistry, our understanding of the ocean carbonate system has come largely from discrete measurements on repeat hydrography cruises that occupy transects across the ocean basins approximately every decade [21–23], monthly to seasonal time-series stations at single locations [7], and underway surface observations of *p*CO_2_ from research vessels and ships of opportunity [24]. These programs have provided invaluable knowledge such as the ability to quantify anthropogenic carbon inventories of the global ocean [2], mean annual oceanic air-sea CO_2_ flux [25], and open-ocean acidification rates [7]. However, there are limitations for ship-based observing strategies. For example, large areas of the ocean have never been sampled due to long transit times and the expensive operating costs of research vessels. Decadal observations provide no information on seasonal or interannual variability. Data can be skewed towards summer months, as research cruises are more frequently conducted during calmer months especially in regions like the Southern Ocean where harsh wintertime conditions make shipboard operations difficult. Capturing the dynamic spatiotemporal variability in coastal oceans can also be challenging from shipboard measurements. In order to meet future scientific and societal needs, development of new observational strategies is required.

The recent expansion of autonomous platforms such as moorings, profiling floats, underwater gliders, and mobile surface vehicles provides a scalable solution to this undersampling problem. Moorings are floating buoys anchored to the bottom of the ocean for typical deployment lengths of up to a year. Profiling floats are buoyancy-driven drifters that sample the water column on a regular cycle, staying in deep waters in between profiles to conserve battery power and limit bio-fouling [26]. Steerable profiling vehicles can be buoyancy-driven underwater gliders [27], or propeller-driven autonomous underwater vehicles [28], with tradeoffs between deployment length and speed. Mobile surface vehicles are relatively new tools for biogeochemical observations, powered by wind or wave energy to achieve comparatively fast speeds while carrying larger payloads than floats or gliders. While observations of *p*CO_2_ from moorings are robust and established, deployments of sensors that measure carbonate system parameters have only recently begun on other autonomous platforms. In particular, the recent addition of pH to profiling platforms has set the stage for revolutionary developments in this field. For example, as of January, 2019, over 10,000 pH profiles (biogeochemical-argo.org) have been made from profiling floats since the first deployments in 2012. This is over twice the number of ship-based pH profiles spanning 1972–2013 [29]. Autonomous platforms and vehicles provide finer scale resolution and coverage than ships or moorings can provide and will form an important part of our future ocean carbonate observing system.

**Fig. 1:**
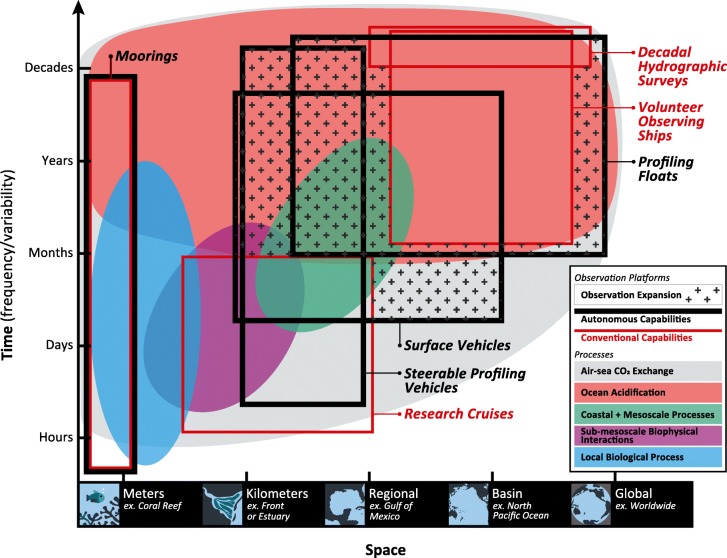
Observational capabilities and carbonate system processes as a function of time and space. Ocean processes that affect the carbonate system (solid colored shapes with labels in the caption) are depicted as a function of the temporal and spatial scales over which they must be observed to capture important variability and/or long-term change. The ability of different platforms to capture carbonate system processes is overlaid for conventional approaches (red boxes, thinner lines) and autonomous arrays (black, thick lines). Not all observational platforms currently provide equivalent measurements capabilities, in terms of either parameters measured or spatial/temporal resolution. For example, profiling floats are only equipped with pH sensors at present, while the Volunteer Observing Ships provide only underway surface measurements. Furthermore, the capability of a given platform to provide long-duration measurements is not entirely captured in this figure; a research cruise may provide a snapshot of a mesoscale process over several weeks, but does not typically capture that process repeatedly over time. The exact spatial and temporal sampling area covered by each platform will change as arrays develop and mature; we have attempted to indicate the spatial sampling coverage likely over the next 5 years. Note that the mooring box includes both open-ocean observatories and compact, fixed observatories deployed in coastal and benthic regions. Box boundaries that are directly adjacent to one another (i.e., the upper boundaries of Profiling floats, Decadal Hydrographic Survey, and Volunteer Observing Ships) indicate the same temporal or spatial boundary but are offset for clarity

In order to distinguish long-term changes in processes from variations in the natural mean state, it is necessary to observe processes over the relevant temporal and spatial scales [30]. For instance, patterns of air-sea CO_2_ exchange are important to observe on short time and space scales in order to understand the gas exchange component of surface observations, but also must be measured globally over annual and decadal time scales to understand long-term changes in the oceanic uptake of anthropogenic CO_2_. Autonomous platforms inherently involve tradeoffs in lifetime, payload, power consumption, and measurement frequency, meaning that no single tool can adequately sample all of the various processes of interest (Fig. 1). For most processes, fully capturing the relevant scales of variability and change will require a mix of vehicles. For example, individual floats can be interpreted in a 1-dimensional sense, providing detailed information on gas exchange and upper ocean processes, while an array of floats, moorings, and gliders can provide information on basin to global scales.

In this review, we summarize work from the past 5 years highlighting the expansion of our autonomous carbonate observing capabilities, and some of the key recent scientific discoveries. Recent reviews have highlighted advances in carbonate sensor technology [31, 32] so we instead focus on the autonomous instrumentation that currently comprises our carbonate observation network and promising emerging technologies. In this paper, we review (1) current autonomous observational capabilities, (2) emerging sensors and autonomous platforms for carbonate observations, and (3) challenges for the measurement and interpretation of these novel observations.

## Current Autonomous Observational Capabilities

### Surface CO_2_ Measurements

Surface moorings are the oldest platform for autonomous observation of the carbonate system. Moorings provide high-frequency observations that can be extended for years to decades, making them suitable to observe variability and long-term changes in ocean chemistry at a given location (Fig. 1). Moorings are a relatively large-capacity platform that can be serviced regularly, allowing for the deployment of complex systems with minimal size and energy constraints. The most common and widespread carbonate system observations on moorings are of *p*CO_2_ by the Moored Autonomous *p*CO_2_ (MAPCO_2_) system [33]. Originally developed from the technology used on shipboard underway systems [34], the MAPCO_2_ system sequentially measures atmospheric and seawater *p*CO_2_ using a nondispersive infrared detector. These measurements yield the *p*CO_2_ difference between air and water (Δ*p*CO_2_) with an uncertainty of ± 2 μatm [33]. A key characteristic of this system is the ability to conduct frequent in situ calibrations using traceable standard gases to achieve climate-quality *p*CO_2_ data when combined with careful pre- and post-deployment quality control and calibration [33].

MAPCO_2_ systems are deployed in a global network at over 40 locations, ranging from open ocean sites to the coastal ocean, with the oldest records spanning up to 15 years (www.pmel.noaa.gov/co2/, [35, 36]). These moorings record year-round observations at most sites, characterizing subseasonal to interannual, as well as regional variability of *p*CO_2_ in an array that can be used to understand the large-scale processes that influence air-sea carbon dioxide fluxes. For example, long-term observations in a tropical coral reef system demonstrated the importance of various processes that influence air-sea CO_2_ flux including rainfall, nutrient delivery, winds, and local biological communities [37, 38]. Sustained mooring observations in the Tropical Pacific revealed that climate forcings such as El Niño/La Niña cycles [35, 39] and anomalous warming events in the Pacific [40] are dominant drivers for interannual variability of *p*CO_2_. Furthermore, these surface observations of *p*CO_2_ are compiled along with shipboard observations into the Surface Ocean CO_2_ Atlas (SOCAT) [24], providing crucial information about the seasonal cycle for calculating global air-sea CO_2_ fluxes (e.g., [41, 42]).

In addition to using surface observations of CO_2_ to quantify large-scale air-sea fluxes and understand the ocean’s role in the global carbon cycle, surface CO_2_ observations have led to an increased understanding of biogeochemical processes in the upper ocean. In order to understand biogeochemical processes, a second carbonate parameter is needed to fully constrain the carbonate system. pH sensors have been developed for moorings (e.g., [43]) but the strong covariance of *p*CO_2_ and pH means that uncertainties in either measurement translate to large uncertainties in the other calculated carbonate system parameters that greatly exceed the uncertainty from using two parameters that covary less strongly [19, 44, 45]. Empirical algorithms relating TA to commonly measured surface variables have allowed the use of single carbonate system parameters such as *p*CO_2_ to yield new understanding of surface carbonate chemistry. For example, in a pair of papers, Fassbender et al. [19, 20] used mooring observations from Ocean Station Papa (Gulf of Alaska) and the Kuroshio Extension to decompose surface biological production into its organic and inorganic components. Similarly, the equatorial *p*CO_2_ observations used to characterize the relationship between *p*CO_2_ and El Niño/La Niña were combined with an algorithm estimate for TA to determine that pH in the region was more variable and changing faster than expected [35]. In coastal regions, MAPCO_2_ measurements off the Eastern coast of the United States were used to show that riverine input and local biological production and respiration were strong drivers of seasonal cycles in *p*CO_2_ [46], while annual coral reef calcification rates were estimated for a rim reef near Bermuda [47].

While the MAPCO_2_ network is providing highly accurate observations and playing a critical role in our understanding of the global ocean carbonate cycle, there are some limitations. For example, moorings cannot provide spatial context to the temporal variability they observe. Furthermore, the costs associated with maintaining and servicing the moorings, especially in the open ocean, make it unlikely that we will be able to significantly increase the size of the mooring array. Finally, subsurface processes greatly affect surface carbonate chemistry; thus, while surface variability can be observed from MAPCO_2_ sensors on moorings, the technology employed on these buoys requires frequent in situ calibration using standard gases, which makes it unsuitable for subsurface measurements.

### Compact, Fixed Observatories

In recent years, a number of in situ sensors for carbonate chemistry have become commercially available, making autonomous measurements more accessible to the community [31]. These sensors allow for routine deployments in a wide range of ecosystems by research groups that are not necessarily experts in instrumentation. We make a distinction from the surface mooring *p*CO_2_ systems in the previous section, as these sensors are smaller (i.e., can be carried by a single person), can make subsurface measurements, and do not require a large surface mooring for deployment.

pH and *p*CO_2_ sensors are available from multiple vendors that utilize a range of sensing techniques [31]. The International Ocean Carbon Coordination Project (www.ioccp.org) maintains an online database with the current status of technology for carbonate chemistry instrumentation. The development of pH sensors based on the Honeywell DuraFET Ion Sensitive Field Effect Transistor (ISFET) technology is arguably one of the most significant recent advancements in autonomous carbonate chemistry measurements. The DuraFET was originally designed for industrial applications [48], but was adapted for oceanographic use after demonstrating excellent stability and performance in seawater [49]. Nernstian behavior over large ranges in pH and salinity was observed, allowing for accurate measurements of pH [50]. The DuraFET was first adapted for shallow water applications [51], and was further modified for high-pressure, profiling float applications [52]. The details and applications for the latter will be presented in the next section. In this section, we highlight studies that utilized self-contained autonomous sensors in fixed locations to investigate carbonate dynamics, with an emphasis on coastal systems.

Nearshore coastal ecosystems are among the most productive in the world and are significant contributors to biodiversity [53]. They also provide huge societal benefits through storm protection and water quality improvement, and provide billions of dollars in revenue from fishing, recreation, and tourism [54]. Many organisms that are thought to be vulnerable to ocean acidification reside in these habitats, such as bivalves, corals, and calcifying algae [10]. Coastal ecosystems are highly dynamic, and can experience natural variability on timescales ranging from hours to interannual (Fig. 1). The magnitude of this natural variability can in some cases be significantly larger than the expected changes due to ocean acidification [55]. High biomass and productivity, a shallower water column, and more pronounced changes in physical conditions contribute to high-frequency variability, which is difficult or impossible to capture using discrete sampling approaches [56].

Compact fixed observatories are particularly useful in characterizing high-frequency variability, and helped guide ocean acidification research in investigating the impacts of natural variability. For example, Hofmann et al. [55] compiled pH sensor data from ecosystems ranging from Antarctica to tropical coral reefs, and presented distinct biome-specific pH patterns that occur on diel, semi-diel, and stochastic timescales. This led to further investigation into exploring patterns of variability in ecosystems such as kelp forests [57, 58], coral reefs [59–62], seagrass meadows [63, 64], continental shelf [65], and upwelling regions [66, 67]. Diel pH variability in coral reefs was found to correlate with community structure and net accretion rates [68], suggesting the potential for natural variability to influence impacts of ocean acidification. Such observations are driving studies to examine the role natural variability plays in organismal response in ocean acidification experiments [69–72].

High-resolution coastal data can also be used to model future carbonate conditions using habitat-specific ocean acidification models. For example, the first high-frequency near-shore record of pH from under sea ice in Antarctica was used to model future wintertime pH [73]. Takeshita et al. [67] decomposed CO_2_ variability into its natural and anthropogenic components and used different atmospheric CO_2_ pathways to model future conditions over an upwelling shelf. The full carbonate system was reconstructed and projected to the end of the century by combining sensor time series and a mechanistic model for a seagrass bed [64]. Development of autonomous systems that can directly measure key fluxes such as benthic metabolism [74, 75] or air-sea flux [76] will help in properly parameterizing such models [77]. These model outputs can act as another guide for experimental conditions in ocean acidification studies of ecosystem responses, a crucial complement to large-scale open-ocean observing systems.

### Global Ocean Observations from Profiling Floats

Profiling floats are the only autonomous observational platform that has been demonstrated to be scalable to a global level for any measurement, and are particularly suited to study basin-wide to global processes on seasonal to interannual timescales (Fig. 1). The Argo profiling float array currently consists of approximately 4000 floats, returning temperature and salinity profiles from 2000 m to the surface every 10 days from around the globe [26]. Given the ability of a float array to make observations on seasonal to interannual timescales at basin to global scales, significant effort has been devoted to integrating biogeochemical sensors onto profiling floats, including oxygen [78, 79], nitrate [80], and bio-optical measurements for chlorophyll *a* fluorescence [81] and particle backscatter [82]. These biogeochemical profiling floats have been used to study production dynamics [83–87], nutrient delivery to oligotrophic waters [88], oxygen minimum zone dynamics [89], regional air-sea fluxes [90–92], and elemental ratios [93]. A *p*CO_2_ sensor has been integrated onto a profiling float [94], but has not left the prototyping phase due to issues such as long response time, need for frequent recalibration, and high power requirements. An ISFET-based pH sensor represents the most recent addition to the sensor suite available for biogeochemical profiling floats and has been demonstrated to be robust and stable throughout the depth range and lifetime of floats [52].

Until recently, most biogeochemical profiling floats were deployed in small numbers by individual researchers or small groups. Building on the success of these individual programs, the Southern Ocean Carbon and Climate Observations and Modeling (SOCCOM) program began the first attempt at creating a biogeochemical float array at the basin scale [95]. The Southern Ocean plays a disproportionate role in moderating the climate through heat uptake, anthropogenic CO_2_ uptake [96], and nutrient delivery to the thermocline [97], yet remains chronically under-sampled due to its remoteness and harsh conditions, especially during the Austral winter. To address this, the SOCCOM project began deploying biogeochemical profiling floats in 2014 with the goal of establishing an array of 200 floats over 6 years [95]. The floats are equipped with oxygen, nitrate, pH, and bio-optical sensors, and provide measurements every 10 days. Currently, there are over 120 pH-equipped floats operating in the Southern Ocean (soccom.princeton.edu), demonstrating that technological challenges have been overcome to operate large biogeochemical float arrays. After post-deployment quality control, (described in the “Challenges” section), these float pH measurements show excellent agreement with independent bottle samples collected at the time of deployment to + 0.005 ± 0.01 (*n* = 952 bottle samples; updated from Johnson et al. [95]).

The array of biogeochemical floats in the Southern Ocean is providing novel insights into air-sea CO_2_ fluxes and biogeochemical processes. For example, large discrepancies in winter surface *p*CO_2_ between float observations and climatologies based on shipboard observations [25] are consistently observed [98–101]. This is not particularly surprising because wintertime shipboard data are sparse. However, the implications for this discrepancy are potentially immense. An initial analysis of 35 floats over 3 years in the Southern Ocean found an annual uptake of only 0.08 ± 0.55 Pg C year^−1^, instead of the ~ 1 Pg C year^−1^ uptake calculated from ship-based estimates, due primarily to increased wintertime outgassing around the Polar Front [100]. This discrepancy represents approximately 50% of the annual contemporary global oceanic CO_2_ uptake [1], and would pose a major challenge to our understanding of the global carbon budget by effectively eliminating the role of the Southern Ocean as a carbon sink. However, a follow-up study combining the float observations with the mooring and underway *p*CO_2_ dataset (SOCAT) indicates that while the outgassing observed by the SOCCOM floats is real and significant, it likely represents a more modest reduction in the Southern Ocean carbon uptake (Bushinsky et al., *in review*, Global Biogeochemical Cycles).

Float-based estimates of other carbonate system parameters can be used in conjunction with biogeochemical sensor data such as nitrate or oxygen as an additional constraint on biogeochemical processes. Williams et al. [99] combined SOCCOM float-derived carbonate system estimates and nitrate data to decompose the seasonal drivers of the carbonate system, finding that carbonate system seasonal cycles agree well with previous climatologies in the spring and summer months but differ in winter months when data were previously sparse. Measurements of biogeochemical parameters under sea ice have been particularly lacking, and this is especially true for carbonate observations. Estimated DIC from under-ice floats was used to help quantify under-ice heterotrophy, but yielded intriguing stoichiometric ratios between inorganic carbon, oxygen, and nitrate that warrant more exploration [102]. These studies highlight the large knowledge gaps that could only be revealed through an array of sustained, autonomous observations.

## Emerging Technologies

In this section, we discuss the likely platforms and sensors that will comprise our near- and long-term autonomous future in observing the carbonate system. One notable development currently underway is the transition of relatively mature carbonate observing sensors from moorings and floats to gliders and autonomous surface vehicles, which involves repackaging of systems rather than development of new sensors. We also discuss new sensing technology for carbonate sensors and the opportunities they might bring.

While moorings and an ever-increasing number of profiling floats are sampling the open ocean and small-scale local observatories have been deployed in many near-shore locations, there is currently a gap in autonomous carbonate observations of regional processes. Coastal regions, boundary currents, and other meso- and submesoscale processes all have significant importance to the global carbon cycle but currently lack sustained autonomous observations for carbonate chemistry [103]. These regions have higher spatial and temporal variability than can easily be sampled by ships and are either too shallow or have fast moving currents that limit sampling by profiling floats. For example, the California coastal upwelling region is both an important fishery and likely to experience early effects of acidification. Cruise transects have observed significant seasonal upwelling-driven corrosive waters (Ω < 1) on the shelf [104] and some areas experience persistent undersaturated conditions [105]. Coastal glider transects using buoyancy-driven vehicles in the California Current System, as part of the California Cooperative Oceanic Fisheries Investigations (CalCOFI) have been used to calculate Ω [106] using empirical relationships with temperature and oxygen [107], finding periodic undersaturation in nearshore waters.

Underwater gliders have been demonstrated to be an effective platform at studying submeso- to mesoscale processes such as fronts and eddies, and to connect the coastal ocean to the open ocean [27]. Equipping gliders with carbonate parameter sensors should provide a much more spatially and temporally detailed understanding in these complex and important regions. Developing new, or adopting existing sensing technology for mobile profiling platforms is challenging, as there are significant constraints on size, reagent consumption, power, response time, and requires well-characterized dynamic errors as the platform moves through the water column. Despite these challenges, there are some larger powered autonomous underwater vehicles (AUVs) that have been equipped with *p*CO_2_ sensors [108] and initial test deployments of pH on gliders look promising [109, 110]. Such regions will also likely require integration of overlapping vehicles and platforms to make best use of the tradeoffs in duration, capacity, and sensor capabilities of our available observing capabilities (Fig. 1).

Another powerful platform for carbonate observations are autonomous surface vehicles powered by wind or waves. Vehicles such as Wave Gliders [111] and Saildrones [112] are faster and more mobile than buoyancy-driven gliders but more expensive to operate and typically have shorter deployment durations. A CO_2_ system originally designed for mooring operations [34] and an ISFET pH sensor mounted on a Wave Glider have produced high-resolution observations of surface *p*CO_2_ and calculated air-sea CO_2_ fluxes in Monterey Bay, California [113]. Similarly, Saildrones have been equipped with modified MAPCO_2_ systems [114] and DuraFET pH sensors and are capable of sampling fast moving currents that may be inaccessible to profiling floats or gliders.

The development and refinement of existing sensing technology will be essential to expanding our autonomous observing capabilities. For example, refinement of sensor design and conditioning significantly reduced initial drift for pH sensors on profiling floats [95]. Continuing to improve our current sensor designs for reliability, performance, and reduction in cost is an underappreciated yet important task. Solid state, low power optical sensors for pH and *p*CO_2_ that use similar sensing principles to the successful and widespread O_2_ sensor have shown promising results in the laboratory and on autonomous platforms [115, 116]. The development of robust, autonomous measurements for additional parameters such as DIC, TA, or carbonate ion concentration ([CO_3_^2−^]) is also highly desired. While using empirically estimated TA is appropriate for some situations, this approach will not work in many coastal systems such as estuaries and coral reefs. Prototype in situ measurement systems for DIC [45, 117–119] and TA [120] have been successfully deployed with encouraging results. However, all of these approaches utilize multiple pumps and valves, making power consumption, size, robustness, and reliability major challenges to overcome. Development of “lab-on-chip” devices has made great strides toward conducting in situ chemistry on microfluidic scales [121], and could provide a path forward for such in situ analyzers. Successful deployments of lab-on-chip devices on underwater gliders have been demonstrated for nitrate [153]. Innovative TA sensors that generate hydrogen ions in situ through coulometry [122] or ion-selective membranes [123] to conduct chronopotentiometric titrations have also been demonstrated in the laboratory. These sensors are small, solid state, and low power, making them promising candidates for in situ sensing applications.

Recently, a method for accurate [CO_3_^2−^] measurement based on spectrophotometry has been developed [124–126]. [CO_3_^2−^] measurements are particularly useful when the primary target is saturation state. The measurement principle is very similar to spectrophotometric pH, and thus should be adaptable for autonomous applications. [CO_3_^2−^] is used to calculate the saturation state of calcium carbonate (Ω) and the ability to measure [CO_3_^2−^] adds a fifth “master” variable to evaluate the carbonate system [4, 125]. This measurement could become a new and exciting tool to monitor ocean acidification [127, 128], especially in coastal areas where low saturation state is thought to be the primary driver for deleterious impacts from ocean acidification for organisms such as bivalve larvae [13, 129] and pteropods [130].

The amount of particulate carbon produced in the form of calcium carbonate relative to primary production of organic carbon is an important but poorly constrained component of the carbonate cycle [131]. Profiling floats have been equipped with optical sediment traps to estimate vertical particle fluxes out of the upper ocean. Initial results indicate these can be converted into carbon flux estimates [132]. The highly specialized Carbon Flux Explorer profiling floats can observe sinking particles and determine particulate inorganic carbon rain rates in addition to organic carbon, finding significantly greater export in the wintertime than indicated by more basic sediment traps [133]. These technologies may provide an additional constraint on estimates of inorganic carbon production from measured carbonate system parameters.

## Challenges

In addition to the development of new sensing technology, similar amounts of effort should be devoted to the development, adoption, and validation of robust calibration protocols. Calibration protocols are essential for successfully operating a network of autonomous platforms, as they ensure accuracy and consistency throughout the array, and sensor drift can be identified and corrected. Such a protocol has been established and implemented for pH on profiling floats. The conductivity sensor (used to calculate salinity) on Argo floats is corrected using deep waters (> 1500 m), as conditions are stable and can be predicted using hydrographic measurements [134]. Following this approach, pH sensors are corrected by comparing sensor pH to a deep reference pH field at 1500 m [95]. The reference pH field is calculated from empirical relationships derived from hydrographic data using temperature, salinity, pressure, and oxygen as inputs [135]. These algorithms can be region-specific [135] or global [136–138]. It should be noted that the accuracy of the reference pH field, and thus corrected sensor pH, depends on other parameters measured on the float such as temperature, salinity, pressure, and oxygen [98]. Therefore, the quality of calibration for the other sensors will affect the final corrected pH data as well. Furthermore, regions where anthropogenic carbon has penetrated into the deep ocean such as the North Atlantic will require regular updates to the algorithms, or inclusion of more robust temporal trends in the algorithm [136]. The quality of float pH data is similar to those from state-of-the-art hydrographic cruises, demonstrating the capability of accurately observing carbonate chemistry from autonomous profiling arrays.

Validation of the SOCCOM calibration protocols has been achieved through the hydrographic casts and discrete bottle data that typically accompany each float deployment. While this has been instrumental for the development and validation of quality control protocols [95], it is not feasible to accompany every biogeochemical float deployment with a full cast of discrete bottle samples. Thus, it will be important to prepare for deployment strategies without validation samples, while continuing to assess the performance of this quality control protocol in different ocean basins. Furthermore, data adjustment based on deep reference pH fields is not always possible, such as for moorings, shallow water process studies, or small-scale observatories. Integrating in situ calibration functionality using certified reference materials such as tris buffer for pH [139] will allow for consistent and accurate datasets across multiple platforms and research groups. As more sensing technologies continue to become available, establishment of best practices [51] to ensure proper calibration, quality control, and sensor intercomparability will be essential.

In addition to calibration of individual carbonate sensors, it is essential that the uncertainties in the estimated carbonate variables are critically assessed. For example, a careful bottom-up uncertainty analysis yielded an estimate of ± 2.86% uncertainty in the SOCCOM float-based *p*CO_2_ estimates, equating to approximately ± 11.4 μatm at 400 μatm *p*CO_2_ [98]. This represents the absolute accuracy of a single float, but the ability of pH-derived pCO_2_ to track spatiotemporal patterns over, e.g., a seasonal cycle is significantly better and is estimated to be ± 5.5 μatm [140]. However, the seasonal to multi-annual long-term stability of surface *p*CO_2_ estimates has not been characterized. No studies have investigated in equal detail possible biases in estimates of DIC or Ω. Furthermore, it is important to distinguish between systematic biases that affect the entire array in the same direction and random uncertainties in individual observations or sensors that are uncorrelated and will average out over the fleet. Small systematic biases in estimated *p*CO_2_ can have a large impact when scaled up to global air-sea flux estimates. The average difference between shipboard underway *p*CO_2_ and float-estimated *p*CO_2_ is 3.7 μatm *p*CO_2_ (*n* = 35–39) [99, 100]. However, it is unclear whether or not this represents a real systematic bias in the float estimates, or an artifact due to small sample size and spatiotemporal variability (± 1 day and ± 25 km) between float and ship measurements. Increased efforts to validate float *p*CO_2_ estimates and determine the magnitude of any bias are crucial as these biogeochemical Argo float deployments expand.

Several issues have been identified in our current thermodynamic model of the marine carbonate system. A recent analysis demonstrated that uncertainty in carbonate system equilibrium constants was a dominant source of error when other parameters in the carbonate system were calculated [141]. For example, a pH-dependent bias between directly measured pH using spectrophotometry and pH calculated from TA and DIC has been identified [136], though the scope and nature of the bias is still under investigation by the community. This has implications for how to calibrate pH sensors using deep values, and its associated uncertainty when estimating surface *p*CO_2_ [98]. These thermodynamic parameters are characterized through careful laboratory experiments and are not as well defined for some of the less commonly sampled waters such as in sea ice regions and coastal waters. In the case of sea ice, where waters are routinely below 0 °C, commonly used carbonate system constants do converge, but brackish coastal waters may require more work [142, 143]. Coastal waters may also contain an unknown contribution of organic alkalinity that is not parameterized in the inorganic carbonate model [144, 145]. These results have highlighted the need for more research into understanding where the uncertainties in the carbonate system may lie [146].

As introduced earlier, float pH measurements can be combined with empirical algorithms of TA to calculate other carbonate parameters. Recently, two new algorithms have been developed to estimate TA and other carbonate system parameters on a global scale based on the same global ocean discrete bottle sample dataset [29]. The LIR (Locally Interpolated Regression) uses a multiple linear regression approach, and interpolates the coefficients of the regression model to any location [136, 147], providing a smooth transition between region-specific relationships. The second approach is CANYON (CArbonate system and Nutrients concentration from hYdrological properties and Oxygen using a Neural-network), which uses a neural network [137, 138]. Both approaches are capable of estimating TA with uncertainties of about ± 6–8 μmol kg^−1^ globally, though a detailed comparison between these two algorithms has not been performed and region-specific algorithms may still be necessary [98]. These global algorithms are an enabling step for a global profiling float array and other autonomous platforms as they provide a framework to calibrate future sensors as the array continues to expand.

Until widespread observations of multiple carbonate parameters are possible, the community will likely continue to rely on algorithm and mapping approaches for carbonate system parameters. Simultaneously measuring two carbonate parameters would alleviate the need to rely on algorithm approaches to estimate TA, but would not remove the need to interpolate and extrapolate from available observations to provide spatially resolved maps. While improvements in mapping methods (e.g., [41, 138]) are an essential component in understanding the oceanic CO_2_ flux on annual to decadal timescales, these products still suffer from any biases that exist in the underlying observational dataset and will require the on-going addition of in situ data to maintain their utility. Just as new profiling float-based estimates of *p*CO_2_ indicate that the interpolation schemes used for surface observations of *p*CO_2_ cannot reproduce a signal they do not observe, it is likely that issues will arise with the use of empirical algorithms for TA and other carbonate system parameters. For example, in regions with highly variable CaCO_3_ production, empirical algorithms based on temperature, salinity, and oxygen alone will not necessarily capture changes in TA associated with calcification and dissolution. Additionally, these algorithms must be continually updated with new training data as surface conditions change due to acidification and natural interannual or decadal variability. Therefore, high-quality ship-based observational programs such as the repeat hydrography and time series programs are critical and will remain a fundamental component of our autonomous future.

The expansion of autonomous observations of the carbonate system should be matched with an increased ability to use and interpret these new measurements. While the makeup of our developing observing system is still in flux, it is clear that the network will be a mix of stationary and mobile platforms that produce both direct measurements and derived quantities. Integration of these autonomous observations with conventional shipboard measurements for use in data synthesis and modeling approaches will be key to leveraging our existing and future capabilities for maximum benefit.

Continued advancement of data management is required to deal with the increasing volume and types of data to make it accessible to the outside community, especially modelers. These data must be readily available, well-documented, and in a user-friendly format to be useful. For example, providing data in a unified data format and stored in central locations has been crucial to the success of the core Argo array, but addition of biogeochemical parameters presents new challenges in post-deployment quality control and data management. The development of cyber infrastructure to deal with differing data formats, recording and propagation of uncertainties, and making data available for near-real-time assimilation into forecast models will all make these datasets more useful. Coordination between the observation, modeling, and data management communities from the early stages of planning will help with this effort.

A successful example of such coordination between communities has been demonstrated by the SOCCOM project [148]. During the planning stages of the project, Observing System Simulation Experiments (OSSEs) were used to simulate an array of Southern Ocean biogeochemical profiling floats and to examine the reconstruction skill that could be achieved from the fleet [149, 150]. These OSSEs provided critical information about the number of floats required to reproduce observed patterns of oxygen, DIC, and air-sea CO_2_ fluxes. The development of the Biogeochemical Southern Ocean State Estimate (B-SOSE; [151]), a data-assimilating numerical model which seeks to minimize model-observation differences using conserved model dynamics, now provides observationally informed output in a gridded format useful for prognostic model evaluation [152]. Looking ahead, the use of observations in conjunction with models, either through assimilating state estimate models or to validate prognostic models, is a powerful tool for synthesizing existing observations and extrapolating our understanding into the future.

## Conclusions

The past 5 years have seen the expansion of our autonomous carbonate observing system. Existing observations by moorings have become an integral part of the community’s ability to estimate large-scale CO_2_ fluxes which are critical to our understanding of the ocean’s role in climate. Float-based estimates of *p*CO_2_ are supplementing these observations, adding new information about previously poorly sampled seasonal cycles. The impact of new pH sensors deployed on SOCCOM biogeochemical profiling floats has demonstrated how basin-scale measurements of the carbonate system can fundamentally alter our understanding of the carbon cycle. Expansion of this approach globally could be an invaluable addition to our carbonate observing system, complementing high-accuracy shipboard observations and broadening our understanding of the global carbonate system. These large-scale observations have been matched by the increase in small-scale observatories in coastal shelf ecosystems and coral reefs.

While our ability to observe the carbonate system has expanded dramatically in recent years, we are often limited to observations of only one carbonate system parameter at a time. For now, empirical estimates of total alkalinity have provided sufficient information to calculate the entire carbonate system, greatly enhancing the value of existing observations. Looking forward, there are many new sensors in the pipeline that will either enable observation on more autonomous platforms or observation of different carbonate system parameters. Expansion of mature sensors onto glider platforms and fast, mobile surface platforms are especially exciting as the integration of sensors and vehicles is already underway. We are currently in the midst of a massive change in observational capability for the ocean biogeochemical community and close to a future where the most important processes impacting the ocean carbonate system will be observable at high accuracy and at high-resolution spatial and temporal scales.
